# Potential antagonistic relationship of *fgf9* and *rspo1* genes in WNT4 pathway to regulate the sex differentiation in Chinese giant salamander (*Andrias davidianus*)

**DOI:** 10.3389/fmolb.2022.974348

**Published:** 2022-09-20

**Authors:** Jiankang Zhang, Xueping Xia, Ying Zhu, Zitong Lian, Haifeng Tian, Hanbing Xiao, Qiaomu Hu

**Affiliations:** ^1^ Yangtze River Fisheries Research Institute, Chinese Academy of Fishery Sciences, Wuhan, China; ^2^ School of Marine Science and Engineering, Qingdao Agricultural University, Qingdao, China

**Keywords:** *Andrias davidianus*, FGF9, rspo1, antagonize, sex differentiation

## Abstract

Farmed chinese giant salamander (*Andrias davidianus*) was an important distinctive economically amphibian that exhibited male-biased sexual size dimorphism. *Fgf9* and *rspo1* genes antagonize each other in Wnt4 signal pathway to regulate mammalian gonadal differentiation has been demonstrated. However, their expression profile and function in *A. davidianus* are unclear*.* In this study, we firstly characterized *fgf9* and *rspo1* genes expression in developing gonad. Results showed that *fgf9* expression level was higher in testes than in ovaries and increased from 1 to 6 years while *rspo1* expression was higher in ovaries than in testes. *In situ* hybridization assay showed that both *fgf9* and *rspo1* genes expressed at 62 dpf in undifferentiated gonad, and *fgf9* gene was mainly expressed in spermatogonia and sertoli cells in testis while strong positive signal of *rspo1* was detected in granular cell in ovary. During sex-reversal, *fgf9* expression was significantly higher in reversed testes and normal testes than in ovaries, and opposite expression pattern was detected for *rspo1.* When FH535 was used to inhibit Wnt/β-catenin pathway, expression of *rspo1*, *wnt4 and β-catenin* was down-regulated. Conversely, expression of *fgf9, dmrt1, ftz-f1* and *cyp17* were up-regulated. Furthermore, when *rspo1 and fgf9* were knocked down using RNAi technology, respectively. We observed that female biased genes were down regulated in ovary primordial cells after *rspo1* was knocked down, while the opposite expression profile was observed in testis primordial cells after *fgf9* was knocked down. These results suggested that *fgf9* and *rspo1* played an antagonistic role to regulate sex differentiation in the process of the gonadal development and provided a foundation for further functional characterizations. The data also provided basic information for genome editing breeding to improve the Chinese giant salamander farming industry.

## Introduction

The Chinese giant salamander *A. davidianus* is the largest extant amphibian, from aquatic to terrestrial vertebrates, in world with the body length to 1.8 m (Turvey et al., 2019). Due to habitat destruction, wild population were sharply decreased. From 1998, *A. davidianus* was listed as endangered on the China Species Red List. Artificial propagation provide a approach to conserve *A. davidianus* and also generate a new industry. From 2006, commercial sale of F2 generation Chinese giant salamander has been allowed by the Chinese government. Now, commercial breeding and farming of giant salamander has became an important industry. However, male salamander grow faster than female which impeded the development of the industry.

Fibroblast growth factor-9 (*fgf9*) is one of the fibroblast growth factor, which plays an important role in the development of ovarian cancer, bone development, and gonadal differentiation (Blasio et al., 1995; Harada and Akita, 2020). *Fgf9* was originally isolated from a human glial cell line. Based on its activity, it was named glia-activating factor (Plotnikov et al., 2001). Later, further studies found that *fgf9* not only plays an important role in ovarian cancer and bone development but also plays a very important role in mammal sex determination and differentiation (Colvin et al., 2001; Behr et al., 2010). In mice, the lack of *fgf9* disrupts the expression of *sox9* in the XY gonads and up regulates the expression of *wnt4*, preventing the embryo from progressing through the male differentiation pathway and resulting in the phenomenon of sex reversal (Colvin et al., 2001). Transgenic mice with loss-of-function *fgf9* exhibit male-to-female sex reversal, and gain of *fgf9* copy number was found in human 46, a patient with XX sex reversal and disorders of sex development (Li et al., 2020).

Kamata et al. (2004) discovered a gene with a thrombospondin (TSP) type I structure and named it R-spondin (roof plate-specific spondin). R-spondin-1 can bind to specific receptors to activate the Wnt/β-catenin pathway to regulate cell proliferation, differentiation and apoptosis (van Meurs et al., 2011). In mice, inactivation of *rspo1* by homologous recombination led to partial XX sex reversal (Zhang et al., 2011). Expression of *rspo1* in various vertebrate species showed that *rspo1* promote ovarian development (Binnerts and Kim, 2007; Wei et al., 2007; Bagheri-Fam et al., 2008; Kocer et al., 2008; Tomizuka et al., 2008). Human *r-spondin1* (*rspo1*) was considered to be an activating gene in ovarian development. *Rspo1* activated the Rspo1/β-catenin/Wnt signal pathway in the human and mouse reproductive system, especially for the early sex differentiation (Kim and Capel, 2006; Tomaselli et al., 2011; Biason-Lauber, 2012). In human, mutations in *rspo1* gene can lead to complete female-to-male sexual reversal (Parma et al., 2006). The results suggested that *rspo1* is necessary for human sex determination.

Studies shown that in mammals, the *sox9*/*fgf9* and *rspo1/wnt4* signal pathways compete with each other to reach a balance to determine sex differentiation (Piprek, 2009). When the expression of male-biased genes (*sox9*, *fgf9*) is higher than that of female-biased genes (*wnt4*, *rspo1*), the balance of sexual differentiation tilts towards the male pathway, and the gonads develop into testes. Otherwise, the gonads will develop into ovaries (Kim et al., 2006)*.* There is an interactive positive feedback loop between *fgf9* and *sox9* (Kim and Capel, 2006). Studies have found that XY *fgf9/wnt4* and *FGFR2/Wnt4* double mutants develop testes with male somatic cells and germ cells, which indicates that the main role of FGF signal is to inhibit female-promoting genes (Jameson et al., 2012). Whether the gonads differentiate towards the testes depends not only on the expression of male genes (such as *sox9*) but also on the active suppression of female genes (such as *wnt4*) (Jameson et al., 2012). The *R-spondin1/Wnt4/β-catenin* pathway and *foxl2* transcription factor work in a complementary manner to promote ovarian growth and inhibit testicular development (Ottolenghi et al., 2007; Maatouk et al., 2008; Nef and Vassalli, 2009). Through the scattered in-frame missense and splicing mutations of the *MAP3K1* gene, the expression of *WNT/β-catenin/FOXL2* activity are enhanced and the expression of *sox9/fgf9/fgfr2/sry* is reduced to mediate the tilting of the sex determination pathway from male to female balance (Loke et al., 2014).

In the previous study, we have determined the beginning time of sex differentiation at 98 dpf (Hu et al., 2019a) and explore the female-specific marker (Hu et al., 2019b). Additionally, we found high temperature at 28°C could induce the sex reversal from undifferentiated genetic female to male and estradiol induce the undifferentiated genetic male to female (Hu et al., 2019c). In present study, we characterized *fgf9* and *rspo1* expression using qRT-PCR, and determined their expression in undifferentiated and differentiated gonad by *in situ* hybridization. We also study antagonistic relationship of *fgf9* and *rspo1* genes using the Wnt/β-catenin inhibitor (FH535). Furthermore, RNAi technology was used to study the function of *fgf9* and *rspo1* genes.

## Materials and methods

### Tissue collection

The experimental animals (*A. davidianus* Blanchard, 1871; Cryptobranchidae, Caudata) were collected from the salamander breeding base at Zhejiang Yongqiang Chinese Giant Salamander Co., Ltd., (Jinhua, Zhejiang Province, China). The salamanders were killed after anaesthesia by MS222 according to Yangtze River Fisheries Research Institute Care Committee (No. 2013001). According to previous study, we found that gonad of *A. davidianus* began to differentiate on the 98 days after fertilization (Hu et al., 2019a). The developing stage gonads of 62 dpf (days post fertilization), 98 dpf, 130 dpf and 1-, 2-, 3-, 4-, 5-, and 6-years-old (6 years old) giant salamanders in the normal group and sex hormone induction group were collected. Two samples of each tissue were collected, and one was stored in RNAstore Reagent for RNA extraction (Tiangen, DP408); the other part was preserved in 4% paraformaldehyde (pH 7.5) to prepare tissue sections; at the same time, the muscle tissue of each individual above was collected and preserved in absolute ethanol for DNA extraction using the TIANamp Genomic DNA Kit (Tiangen, Beijing, China) including RNase A treatment according the manufacturer’s instructions (Hu et al., 2019b). The testes, ovary, liver, spleen, lung, kidney, stomach, intestine, brain and pituitary tissues were collected from 2 years old *A. davidianus*, and put into RNAstore Reagent, and then transferred to a refrigerator at −80°C for storage until RNA extraction.

### Identification of physiological sex and genetic sex

The larvae at 55 dpf were treated by estradiol or high temperature (28°C) respectively until 8 months post-fertilization to obtain the sex reversal individual according the previous study (Hu et al., 2019c). Using tissue sections and microscopes, the physiological sex of *A. davidianus* samples was identified, and, the genetic sex of used *A. davidianus* were identified using female-specific markers *adf340* developed by our laboratory (Hu et al., 2019b). The PCR system consisted of 12.5 μl 2X Master Mix (TsingKe, TSE003), 100 ng genomic DNA, 0.5 µl of each primer (10 μmol/L), and double distilled water to reach a final volume of 25 μl. The reaction conditions were as follows: 95°C for 5 min; 33 cycles of 94°C for 15 s, 60°C for 15 s, 72°C for 30 s; and 72°C for 5 min. If the sex and genotype were consistent, there was no sex reversal. If the sex and genotype were different, namely, the sex was male, and the genotype was female, the individual was considered a sex reversal male (RM) individual; if the sex was female, and the genotype was male, the individual was considered a sex reversal female (RF) individual.

### Cloning of the *fgf9* and *rspo1* genes

According to the partial sequences of the *fgf9* and *rspo1* genes in the giant salamander transcriptome database from our laboratory (Hu et al., 2019c), primers were designed to amplify the 5′ or 3′ UTR (Table 1). The 5′-RACE-Ready cDNA and 3′-RACE-Ready cDNA libraries were constructed according to the SMART™ RACE kit (Clontech, 634923) instructions as templates for RACE. PCR was performed as follows: five cycles of 94°C for 30 s and 72°C for 3 min; five cycles of 94°C for 30 s, 70°C for 30 s, and 72°C for 3 min; and then 27 cycles of 94°C for 30 s, 68°C for 30 s, and 72°C for 3 min. The amplified product was electrophoresed on 1% agarose and the purified fragment was cloned into the pMD18-T vector, transfected into *E. coli strain TOP10* (Invitrogen, C404003), and then sequenced.

**TABLE 1:** Primers and sequences used in this study.

Primers	Primer sequences (5′-3′)	Utilizations
UPM-long	CTA​ATA​CGA​CTC​ACT​ATA​GGG​CAA​GCA​GTG​GTA​TCA​ACG​CAG​AGT	5′or 3′RACE
UPM-short	CTA​ATA​CGA​CTC​ACT​ATA​GGG​C	5′or 3′RACE
*fgf9*-5′GSP	GGC​CCA​GGT​GCT​CGC​TCA​ACA​ACA​TCG	5′ region clone
*fgf9*-3′GSP	AGG​GCA​TCC​TAA​GGC​GCA​GGC​AGC​TCT	3′region clone
*rspo1*-5′GSP	GGA​ACA​CTT​CAG​GCA​CCC​GTT​GGA​CT	5′ region clone
*rspo1*-3′GSP	TCC​GAG​TCC​AAC​GGG​TGC​CTG​AAG​T	3′region clone
*adf340*s	TTA​ACG​GCC​CTA​ACA​CCA​GG	Sex identification
*adf340*a	GGT​TTA​GGG​CGG​CTC​TGA​TT	Sex identification
*fgf9-*S	TTG​GCA​GCA​TCC​TCG​TTT​G	qRT-PCR
*fgf9*-A	CAG​GTG​CTC​GCT​CAA​CAA​CAT	qRT-PCR
*rspo1*S	GTC​CAA​CGG​GTG​CCT​GAA​G	qRT-PCR
*rspo1*A	ATT​GCG​AAC​GCC​GAA​GTA​TC	qRT-PCR
fzd2-A	TGC​GAT​GGG​TTG​GCA​GTA​G	qRT-PCR
fzd2-S	CTG​AGG​GAG​CAA​AGA​GGG​C	qRT-PCR
Wnt4-A	CTC​GGG​TCC​CTT​GCG​TTA​C	qRT-PCR
Wnt4-S	CGGTGGGCAGCATTTCAG	qRT-PCR
β-catenin-A	GCA​CAG​GTT​ACA​ACA​TTG​ATG​TCA​T	qRT-PCR
β-catenin-S	CAA​ACC​TGC​GAT​TGT​TGA​AGC	qRT-PCR
*β-Actin*-f	GGT​TAT​GCC​CTG​CCT​CAC​G	Internal control
*β-Actin*-r	ATT​TCC​CTT​TCG​GCT​GTG​G	Internal control
*fgf9*-S2	GGT​GGA​AAG​CCC​GAT​GTT​G	*In situ* hybridization
*fgf9*-R2-T7	ATC​ACT​AAT​ACG​ACT​CAC​TAT​AGTGA​TGC​CAC​TTA​GTC​CTA​GTC​CCT	*In situ* hybridization
*fgf9*-S2-T7	GAT​CAC​TAA​TAC​GAC​TCA​CTA​TAGGGT​GGA​AAG​CCC​GAT​GTT​G	*In situ* hybridization
*fgf9*-R2	TGA​TGC​CAC​TTA​GTC​CTA​GTC​CCT	*In situ* hybridization
*rspo1*S2	TCC​TGA​GCT​TCA​TGG​ATG​TAG​C	*In situ* hybridization
*rspo1*R1-T7	GAT​CAC​TAA​TAC​GAC​TCA​CTA​TAGCGT​CGT​GTC​TCC​CGA​AGG​T	*In situ* hybridization
*rspo1*S2-T7	GAT​CAC​TAA​TAC​GAC​TCA​CTA​TAGTCC​TGA​GCT​TCA​TGG​ATG​TAG​C	*In situ* hybridization
*rspo1*R1	CGT​CGT​GTC​TCC​CGA​AGG​T	*In situ* hybridization
ad*fgf9*-siRNA-890	GCA​CCC​GGC​AAG​AUC​ACA​ATT	Sence
ad*fgf9*-siRNA-890	UUG​UGA​UCU​UGC​CGG​GUG​CTT	Antisence
ad*fgf9*-siRNA-965	GCG​UAG​ACA​GUG​GAC​UCU​ATT	Sence
ad*fgf9*-siRNA-965	UAG​AGU​CCA​CUG​UCU​ACG​CTT	Antisence
ad*fgf9*siRNA-1119	GCG​AUA​UUA​UGU​GGC​UCU​ATT	Sence
ad*fgf9*-siRNA-1119	UAG​AGC​CAC​AUA​AUA​UCG​CTT	Antisence
ad*rspo1*-siRNA-551	GCA​AAG​GGU​UGU​GAC​UCU​UTT	Sence
ad*rspo1*-siRNA-551	AAG​AGU​CAC​AAC​CCU​UUG​CTT	Antisence
ad*rspo1*-siRNA-684	GCG​UUC​GCA​AUC​CAG​ACA​UTT	Sence
ad*rspo1*-siRNA-684	AUG​UCU​GGA​UUG​CGA​ACG​CTT	Antisence
ad*rspo1*-siRNA-771	GCA​AAG​AAG​GCU​UGU​ACU​UTT	Sence
ad*rspo1*-siRNA-771	AAG​UAC​AAG​CCU​UCU​UUG​CTT	Antisence
Negative control (NC)	UUC​UCC​GAA​CGU​GUC​ACG​UTT	Sence
Negative control (NC)	ACG​UGA​CAC​GUU​CGG​AGA​ATT	Antisence

The sequence was searched in the GenBank database using BLAST (http://www.ncbi.nlm.nih.gov/BLAST/). The protein molecular weight was deduced using the website (http://www.bio-soft.net/sms/index.html). The conserved domain was predicted with online software (http://www.ncbi.nlm.nih.gov/structure). The protein domains of *fgf9* and *rspo1* were analyzed by the online software SMART (http://smart.embl-heidelberg.de/smart/set_mode.cgi). The phylogenetic tree of *fgf9* and *rspo1* was constructed based on protein sequence by MEGA 7.0 (Kumar et al., 2016) with the NJ method (neighbor-joining method). Bootstrap values were estimated from 1,000 replications.

### *In situ* hybridization

To detect *fgf9* and *rspo1* expression in gonadal tissue, two pairs of primers were designed to obtain a riboprobe (Table 1). A 468 bp cDNA fragment was amplified from the *fgf9* ORF domain, and a 533 bp cDNA fragment was amplified from *rspo1*. Primers were designed to introduce the T7 promoter sequence in the 5′- or 3′- side of the amplicons that will later be used as riboprobes, and PCR products were purified by a QIAquick Gel Extraction Kit (QIAGEN, Cat# 28706). To obtain probes, transcription was performed using the MEGAshortscript T7 High Yield Transcription Kit (Invitrogen, AM1354) according the manufacturer’s instructions *In situ* hybridization was performed following the method described in previous study (Hu et al., 2016). Sections were deparaffinized, hydrated and treated with proteinase K and then hybridized using sense or antisense DIG-labeled RNA probe at 70°C for 12 h. Hybridization signals were then detected with anti-DIG (Roche, 11093274910) conjugated with POD. DAB (Beyotime, P0202) was used as substrate for POD (Hu et al., 2016).

### Isolation of primary gonadal cell and FH535 treatment

Ovary of female at 1 year old were dissected and washed with the PBS containing 1% Penicillin-Streptomycin (P/S) (Gibco, Thermo Fisher Scientific), then the gonads were cut into small pieces and placed on a 70 μm nylon mesh (Biologix,15-1070) and gently pushed through the mesh with constantly dripping of cold DMEM/F-12 medium (Gibco, 11320082) containing 10% FBS (BioInd, 18455), 1% heparin (Gibco, A16198.03) and Penicillin-Streptomycin (P/S) (Gibco, 15140122). The collected cells were washed using DMEM medium and then centrifuged at 500 × g for 10 min at 4°C. The supernatant was discarded and the cells were collected. The cells were divided into six groups and seed in a 6-well plate (Corning) with the density of 1 × 10^6^ cells/well. Three were set as control and the other three for FH535 treatment. The FH535 (MCE,108409-83-2) was added to the treated groups with the final concentration of 20 uM and incubate at 28°C for 3 days. The cells were collected and RNA was extracted for qRT-PCR.

### RNA interference

According to *fgf9* and *rspo1* sequence, three siRNA at different sites were designed and synthesized for each gene (GenePharma, Shanghai, China, Table1). The siRNA marked by FAM was used as the negative control. The primordial gonadal cells were prepared according to the above description. The siRNA was transfected into the gonadal cells according to the manufacture’s instruction of Lipofectamine TM3000 (Thermo Fisher Scientific). Then the cells were incubated in opti-MEM medium (Gibco, Thermo Fisher Scientific) at 37°C for 6 h. After that, opti-MEM medium was removed and 2 ml of DMEM medium containing 10% FBS and P/S were added and incubated at 28°C for 48 h. The signal of green fluorescence was detected by OLYMPUS IX73. The RNA was extracted according the TRIzol method for qRT-PCR. To further detect the function of *fgf9* and *rspo1,* the siRNA of *fgf9* and *rspo1* with the best inhibition effect were injection into the salamander after the sex was identified by the sex specific primers according to previous study (Hu et al., 2019b; 2019d). Sex related gene expression profile were detected in ovary and testis after *rspo1/*siRNA and *fgf9/*siRNA treatment for 72 h, respectively.

### Quantitative real-time PCR

Total RNA was isolated from tissues using Trizol reagent (Invitrogen, 15596018). The cDNA was synthesized using PrimeScript™ RT reagent Kit following manufacturer’s instructions (Takara, RR047A). The expression of *fgf9 and rspo1* genes in various tissues and developing gonad in *A. davidianus* was analyzed by quantitative real-time PCR (qRT-PCR). qRT-PCR was conducted using 2 × T5 Fast qPCR Mix (SYBR Green) (TsingKe, TSE202) following manufacturer’s instructions independently in triplicate on the QuantStudio 5 Real-Time PCR System (Applied Biosystems, California, United States). *β-Actin* was used as the internal control gene, cDNA was used as a template, tissue from three individuals was used, and each reaction was repeated three times. qRT-PCR was performed as follows: 95°C for 30 s; forty cycles of 95°C for 5 s, 60°C for 30 s, and 72°C for 30 s; and then 72°C for 5 min. The difference in expression was analyzed by one-way ANOVA followed by Duncan multiple comparison tests using SPSS 22.0 (IBM, New York, NY, United States). Significance was set at *p* < 0.05. Bars with different letters differ significantly (*p* < 0.05).

## Results

### Cloning and sequence analysis of *fgf9* and *rspo1* cDNA

With RT-PCR and RACE amplification, full-length *fgf9* and *rspo1 cDNA* were cloned from *A. davidianus* gonad at 1 year old (GenBank Accession No. MW685518 and MW685519). The full length of *fgf9* was 1489 bp with a 5′UTR of 612 bp and a 3′UTR of 247 bp, and the full length of *rspo1* was 1708 bp with a 5′UTR of 430 bp, an ORF of 786 bp, and a 3′UTR of 492 bp. The ORF of *fgf9* encoded a protein of 209 aa, and that of *rspo1* encoded a protein of 261 aa, with molecular weights of 23.35 and 28.76 kDa, respectively. A comparison of the selected amino acid sequences of amphibian and mammalian *fgf9* showed that this gene is conserved with more than 85% identity (Supplementary Figure S1A). Based on the alignment of the amino acid sequence, a phylogenetic tree was constructed by the neighbour-joining method. The tree of *fgf9* showed that *A. davidianus* is more similar to *Cynops orientalis* with 93% identity than to *Xenopus laevis* or *Xenopus tropicalis* (Supplementary Figures S1A,B). The phylogenetic tree of *rspo1* showed that *A. davidianus* is more similar to *C. orientalis* with 85% identify than to *Xenopus laevis* or *Xenopus tropicalis* (Supplementary Figure S2B).

### Expression of *f*g*f9* and *rspo1* in various tissues and developing gonads

**FIGURE 1:**
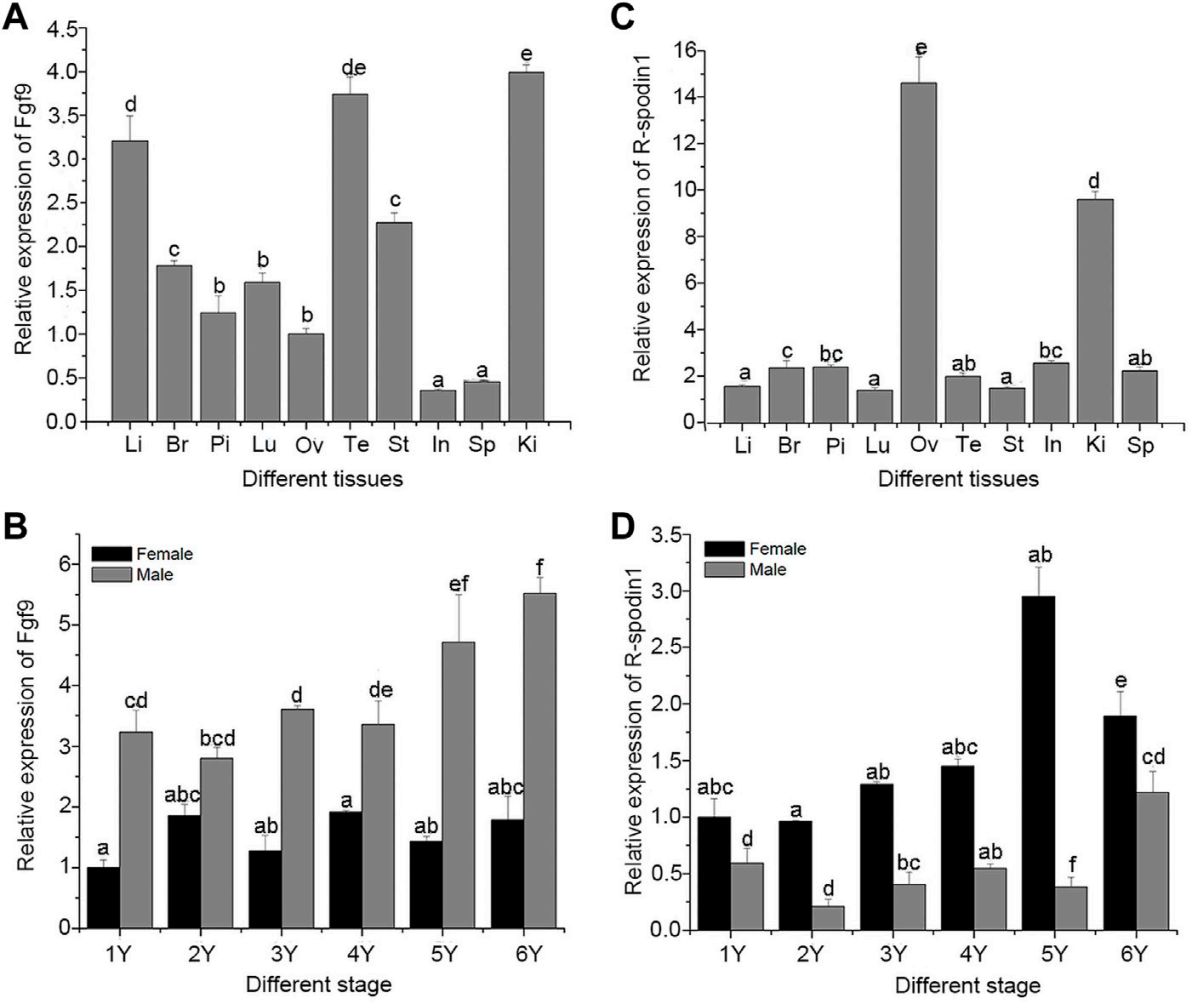
Expression of *fgf9* and *rspo1* gene: expression analysis of fgf9 **(A)** and *rspo1* **(B)** in various tissues by qRT-PCR; expression analysis of fgf9 **(C)** and *rspo1* **(D)** in developing gonads by qRT-PCR. Tissue from three individuals and each reaction was repeated three times. Bars with different letters are significantly different (*p* < 0.05). Li, Liver; Br, Brain; Pi, pituitary; Lu:Lung; Ov, Ovary; Te, Testis; St, Stomach; In, Intestinal; Ki, Kidney; Sp, Spleen.

Genetic sex of the salamander were identified and salamanders without sex reversal were used to analyze the expression profile. Expression levels of the *fgf9* and *rspo1* genes in various tissues from 1 year old and developing gonad were detected using qRT-PCR. High expression levels of *fgf9* were observed in the kidney and testis, followed by the liver, and relatively low expression levels were observed in the spleen and intestine; other tissues showed medium expression levels (Figure 1A). Expression pattern of *fgf9* in developing gonad indicated that the expression of *fgf9* in testes was significantly higher than that in ovaries (*p* < 0.05), and mRNA transcription gradually increased from 1 to 6 years of life (Figure 1B). Expression of *rspo1* was observed in various tissues and significantly higher expression levels were observed in the ovary than that in other tissues (*p* < 0.05), medium expression levels were observed in the kidney, and low levels were observed in the remaining tested tissues (Figure 1C). In the developing gonad, *rspo1* expression was significantly higher in the ovary than in the testis, with expression levels gradually increased from 1 to 5 years of life and decreasing at 6 years (Figure 1D).

### Expression of *fgf9* and *rspo1* identified by *in situ* hybridization

**FIGURE 2:**
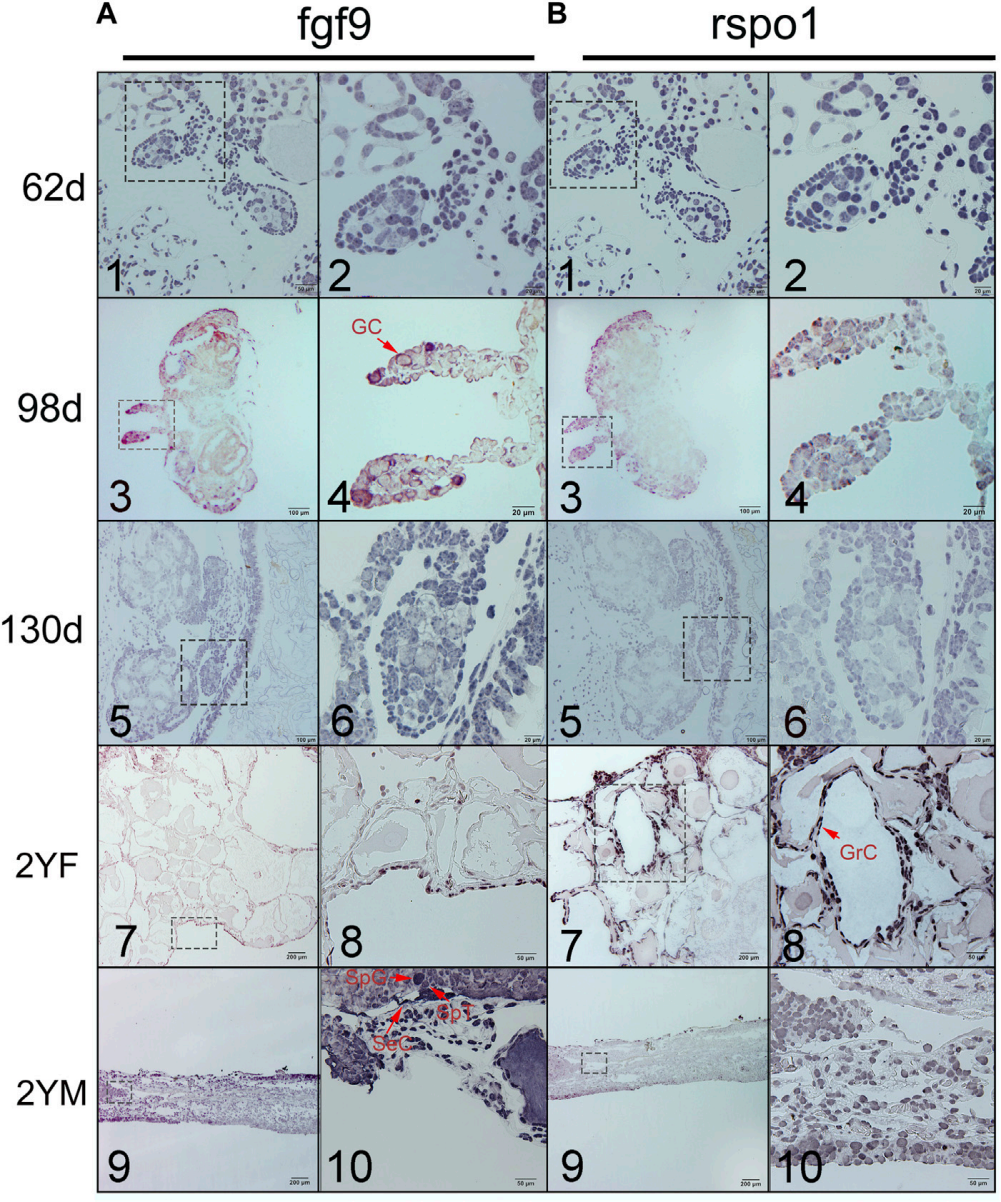
Expression of *fgf9* **(A)** and *rspo1* **(B)** in gonads detected by *in situ* hybridization. 1. Undifferentiated gonad at 62dpf; 2. Large magnification of frame area in (1); 3. Differentiating gonad at 98 dpf; 4. Large magnification of frame area in (3); 5. Differentiating gonad at 130dpf; 6. Large magnification of frame area in (5); 7: 2 year old ovary; 8. Large magnification of frame area in (7); 9. 2 year old testis; 10. Large magnification of frame area in (9). 62dpf: gonad at 62 days after fertilization; 98dpf: gonad at 98 days after fertilization; 2 YF: 2-year-old female gonad;2 YM: 2-year-old male gonad. Three samples were used per stage. Germ cell (GC), granulosa cells (GrC), sertoli cell (SeC), spermatogonia (SpG), spermatocyte (SpT).

To detect whether *fgf9* or *rspo1* expressed before gonad differentiation, *In situ* hybridization was conducted to identify the expression of *fgf9* and *rspo1* in the early developing gonad which was too small to detect by the qRT-PCR. At 62 dpf before the gonad differentiation, *fgf9* and *rspo1* positive signal were detected in the undifferentiated gonad of *A. davidianus* (Figures 2A1,A2,B1,B2). While the sense probe had no signal (Supplementary Figure S3). At 98 dpf when the gonad begin to differentiation, positive signal of *fgf9* and *rspo1* were also both detected in developing gonad (Figures 2A3,A4,B3,B4). At 130 dpf, expression of *fgf9* and *rspo1* were also detected in the gonad. When the gonadal differentiation is complete, we observed that *fgf9* signal was strongly distributed in spermatogonia, spermatocyte and sertoli cells in testis while very weak signal in granular cell in ovary in the 2 years old *A. davidianus* (Figures 2A7,A8,A9,A10). For *rspo1* gene, strong signal was detected in granular cell in ovary and weak signal in spermatogonia, spermatocyte and sertoli cells in testis (Figures 2B7,B8,B9,B10).

### Expression profile of *fgf9* and *rspo1* in sex reversal

**FIGURE 3:**
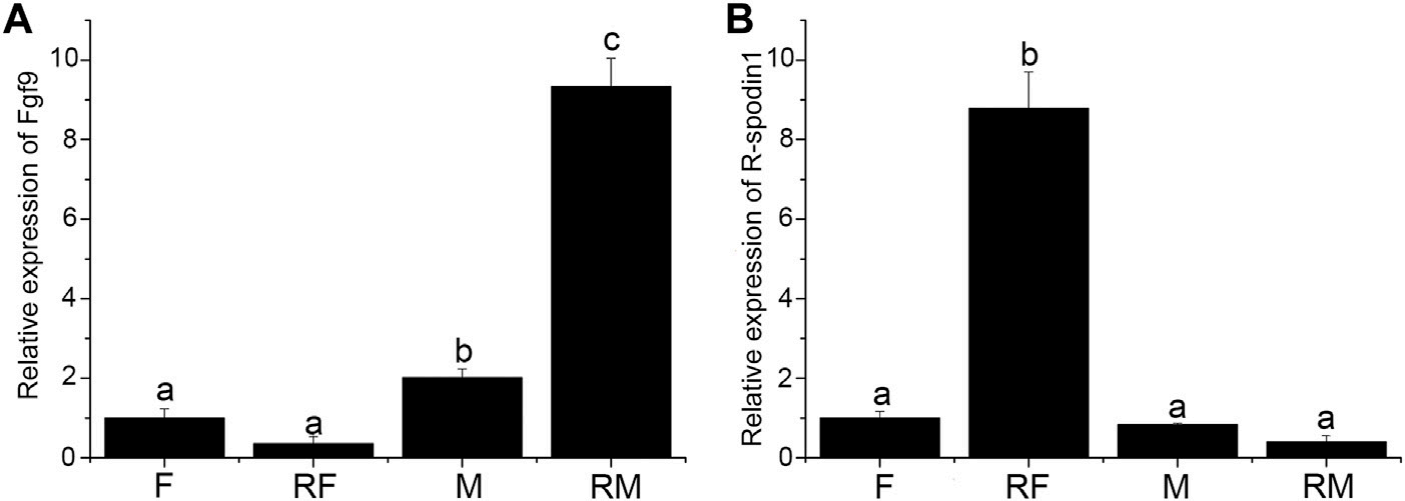
Expression of *fgf9* and *rspo1* in normal gonads and sex-reversal gonads by qRT-PCR. **(A)** Expression analysis of *fgf9* mRNA in normal gonads and sex-reversal gonads by qRT-PCR; **(B)** expression analysis of *rspo1* mRNA in normal gonads and sex-reversal gonads by qRT-PCR. The gonads were collected at 1 year old from three individuals and each reaction was repeated three times. Bars with different letters are significantly different (*p* < 0.05).

Expression level of *fgf9* and *rspo1* were detected in the gonad and sex-reversal gonad at 1 year old. Expression of *fgf9* was significantly higher in testis than in ovary and highest expression was observed in sex-reversal testis (Figure 3A). Conversely, in the gonads and sex-reversal gonads, the expression level of *rspo1* was significantly higher in sex-reversal ovaries than in ovaries, and the *rspo1* expression level was significantly higher in ovaries and sex-reversal ovaries than the testes and sex-reversal testes (*p* < 0.05) (Figure 3B).

### Gene expression profile change after FH535 inhibit Wnt/β-catenin pathway

**FIGURE 4:**
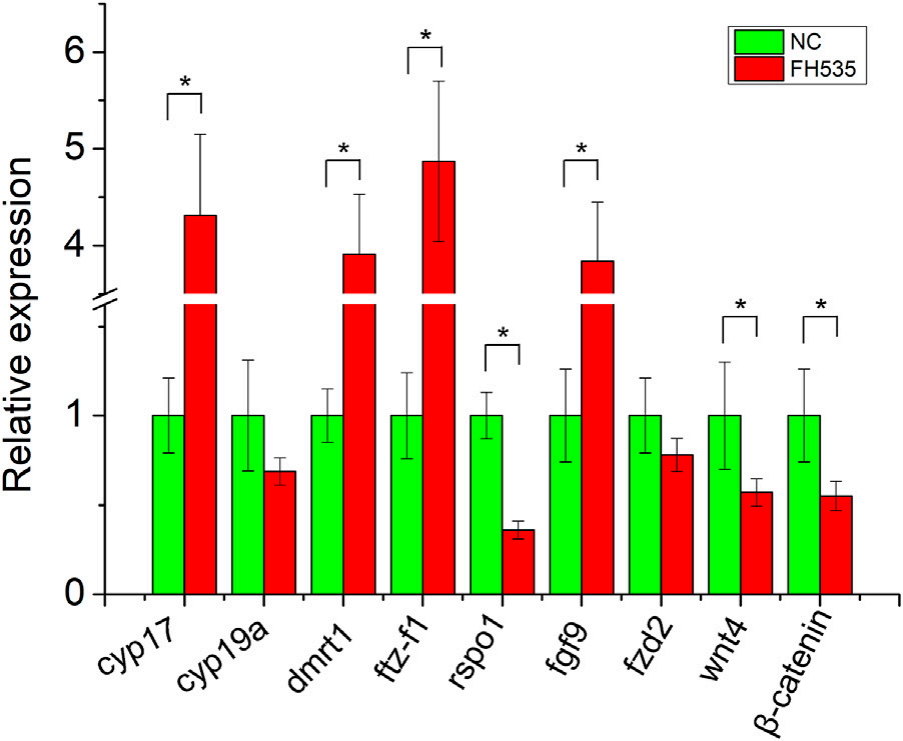
Expression profile of sex-related genes after FH535 treatment. The results from three replicates and *indicate the significantly difference (*p* < 0.05).

To provide further evidence of *rspo1* in gonad development and potential antagonistic relationship of *fgf9* and *rspo1* genes, primary gonadal cell from 1 year old salamander ovary were collected and incubated with or without FH535. After the primary gonadal cells were treated with FH535, expression profile of sex related gene showed that *cyp17*, *dmrt1*, *sf1*, *fgf9* expression were upregulated while *rspo1, wnt4* and *β-catenin* expression was down-regulated comparing with the control without FH535 treatment (Figure 4). Additionally, we observed that *cyp19a* and frizzled2 expression profile was unchanged (Figure 4).

### Gene expression profile after siRNA treatment

**FIGURE 5:**
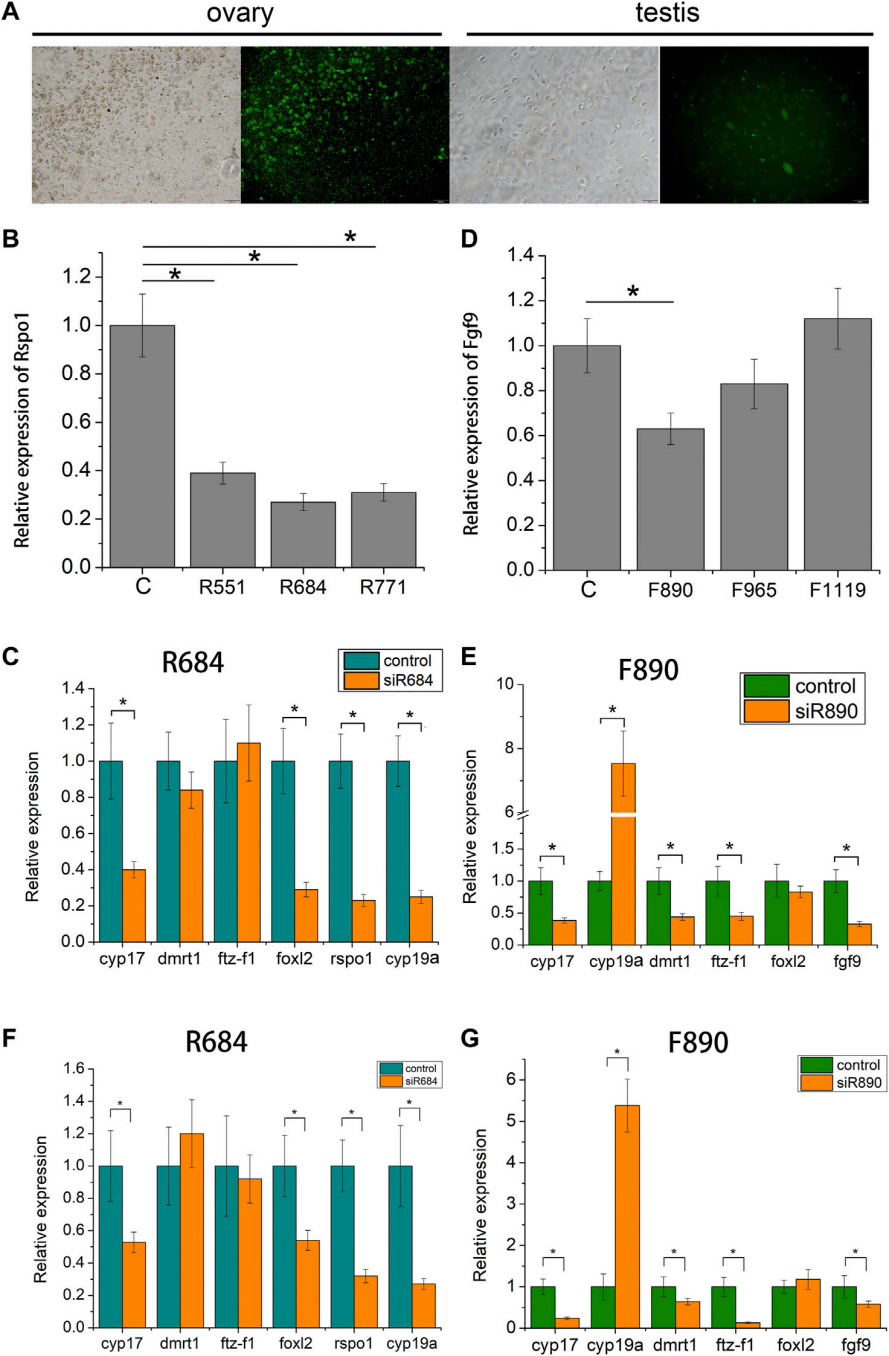
Expression profiles of sex-related gene in *Andrias davidianus* gonad after siRNA treatment. **(A)** control siRNA marked by FAM transfected into the gonadal cells; **(B)** Expression level of each *rspo1* siRNA sites in ovary cell; **(C)** expression profile of sex-related genes after R684 treatment in ovary cell; **(D)** expression level of each *fgf9* siRNA sites in testis cell; **(E)** expression profile of sex-related genes after F890 treatment in testis cell; **(F)** expression profile of sex-related genes after R684 treatment in ovary tissue; **(G)** expression profile of sex-related genes after F890 treatment in testis tissue. The results from three replicates and *indicate the significantly difference (*p* < 0.05).

To examine the function of *fgf9* and *rspo1* genes in the gonad, three siRNA sites were designed according the sequence of *fgf9* and *rspo1,* respectively*.* The control siRNA site marked by FAM was transfected into the gonadal cells and we found that high transfection efficiency was exhibited in ovary cell with 60% and low in testis cell with about 20% (Figure 5A). Expression level of *fgf9* and *rspo1* were significantly down-regulated in ovary and testis cells at site siRNA R684 and F890, respectively (Figures 5B,D). Seventy-two hours post siRNA R684 treatment *in vitro* and vivo, we found that expression of *cyp17*, *foxl2, rspo1* and *cyp19a* were significantly down regulated while *dmrt1* and *ftz-f1* expression were not changed (Figures 5C,F). Seventy-two hours after siRNA F890 treatment in testis cells, expression of *cyp17, fgf9, dmrt1* and *ftz-f1* were significantly down regulated while *cyp19a* expression was up regulated (Figures 5E,G). Only *foxl2* expression was not changed.

## Discussion

Among different *A. davidianus* tissues, the highest expression level of *fgf9* was observed in the kidney, followed by the testis and liver, and a lower level was found in other tissues. In previous studies, *fgf9* has been proven to be widely expressed in various tissues in goats, mice, and cattle (Liao and Lin, 2009; Lai et al., 2016; Huang, 2020). Yamamura found that in *Rana rugosa fgf9* was highly expressed in the ovary but was not detected in the testis; however, it did not show a sex-dependent expression pattern during the sex determination process. Therefore, Yamamura found *fgf9* may not be a key factor in the gonadal differentiation of *Rana rugosa* (Yamamura et al., 2005). In this study, the expression of *fgf9* increased from 1 to 6 years during gonadal development, and the expression in the testis was higher than that in the ovaries of the same stage, indicating that *fgf9* plays a role in testicular development. Additionally, we found that *fgf9* expression was increased during female to male sex reversal which suggested that *fgf9* was associated with testis differentiation. Similar expression pattern was observed in other species. In mice, when *fgf9* was knocked out, the male to female sex reversal was happen (Colvin et al., 2001). In the mouse, *fgf9* is expressed in gonad prior to sex determination, Loss of *Fgf9* leads to XY sex reversal (Kim et al., 2006). The results of *in situ* hybridization showed that *fgf9* was expressed in the undifferentiated gonad of *A. davidianus* at 62 dpf and 98 dpf. Previous study showed that the gonads of *A. davidianus* began to differentiate on the 98 days after fertilization (Hu et al., 2019a), and the *fgf9* gene signal was detected in the gonads of *A. davidianus* at 62 dpf and 98 dpf, indicating that *fgf9* was expressed in *A. davidianus* at the early stage of gonadal differentiation and affected gonadal differentiation. The weakly positive signal in the ovarian granulosa cells of the 2-years-old *A. davidianus* and the strong positive signal in the testis indicate that the *fgf9* gene always plays an important role in the testes of *A. davidianus*, but the role in the ovary is not obvious.

The relative expression of *rspo1* was assessed by qRT-PCR. The results showed that the relative expression of *rspo1* in *A. davidianus* was the highest in the ovary. The level in the ovary was significantly higher than that in other tissues. Similar *rspo1* expression patters were reported in *Peldiscus sinensis* (Zhang, 2019). The expression of *rspo1* in *Danio rerio* was significantly higher in adult females than in males in the kidney, gonad and muscle. During the gonadal differentiation of vertebrates, the expression of *rspo1* in females was significantly higher than that in males. In the developing gonad of *A. davidianus*, the *rspo1* expression level was significantly higher in the ovary than in the testis, with expression levels gradually increasing from 1 to 5 years and decreasing at 6 years. The results showed that *rspo1* gradually played a role in ovarian development, but after sexual maturation, the level of *rspo1* began to decline, and the expression level in testis began to rise, suggesting that it may play some role in sexually mature testis. However, there is no relevant research or report on the function of *rspo1* in testis. The *rspo1* gene can play a key role in human and mouse reproductive systems by activating *rspo1/β-catenin/Wnt* signal pathways, especially in early sex determination (Nam et al., 2007; Zhang et al., 2011). *Wnt4* has been found to promote ovarian differentiation by inhibiting male sex differentiation (Biason-Lauber and Konrad, 2008). But the expression profile of *wnt4* in the ovary was affected by changes of protein R-spondin-1 (Biason-Lauber, 2012). In the present study, we used FH535 to inhibit *rspo1* expression. From the results, we observed that potential antagonistic genes *fgf9* expression was upregulated and expression of sex differentiation gene were changed except cyp19a. The results suggested that *fgf9* antagonizes the expression of *rspo1* gene and regulate sex differentiation through *rspo1/β-catenin/Wnt* signal pathways. Smith found that *rspo1* is a highly conserved and ancient part of the vertebrate ovarian determinant (Smith et al., 2008). The localization of *rspo1* gene expression in ovarian and testicular cells was identified by *in situ* hybridization in *A. davidianus*. The results showed that *rspo1* gene signals were both detected in the gonads of *A. davidianus* at 62 and 98 dpf, suggesting that *rspo1* may play a role in gonadal differentiation and that when the gonads of *A. davidianus* begin to differentiate, *rspo1* begins to be expressed, affecting the gonad differentiation of *A. davidianus*. The *rspo1* gene positive signals were strongly detected in the ovarian cells in 2-years-old *A. davidianus* (Figure 2B7), while the positive signal was not strong in the testis (Figure 2B9), suggesting that *rspo1* affects the process of gonadal differentiation into ovary and that it may also play a role in the development of testis, but this role has not yet been explained. Among the gonads and sex-reversal gonads, the expression level of *rspo1* was the highest in sex-reversal ovaries, followed by ovaries, and the *rspo1* expression level was significantly higher in ovaries and sex-reversal ovaries than that in testes and sex-reversal testes. The above results showed that *rspo1* was highly expressed in ovaries and different tissues and during developmental stages of *A. davidianus*, as well as in gonads and sex-reversal gonads in *A. davidianus*. It can be determined that *rspo1* plays an important role in ovarian differentiation and development.

## Conclusion

In the present study, we characterized fgf9 and rspo1 genes expression in various tissues and developing gonads. *In situ* hybridization was used to detect the expression of fgf9 and rspo1 in undifferentiated gonad and locate the expression. Function and potential antagonistic relationship of *fgf9* and *rspo1* genes were studied and confirmed that potential antagonistic relationship of *fgf9* and *rspo1* genes in WNT4 pathway to regulate the sex differentiation in *A. davidianus.* Furthermore, genome editing technology would carried out for further study in future. The data provided basic information for genome editing breeding to improve the Chinese giant salamander farming industry.

## Data Availability

The datasets presented in this study can be found in online repositories. The names of the repository/repositories and accession number(s) can be found below: https://www.ncbi.nlm.nih.gov/genbank/, MW685518; https://www.ncbi.nlm.nih.gov/genbank/, MW685519.

## Supplementary material

SUPPLEMENTARY FIGURE S1 Multiple alignment of the FGF9 amino acid sequence **(A)** and NJ phylogenetic tree **(B)**.

SUPPLEMENTARY FIGURE S2 Multiple alignment of the RSPO1 amino acid sequence **(A)** and the NJ phylogenetic tree **(B)**.

SUPPLEMENTARY FIGURE S3 Result of in situ hybridization with fgf9 **(A)** and rspo1 **(B)** sense 628 probe; no positive signal was detected.
