# Supplementary figures and images for: Potential antagonistic relationship of fgf9 and rspo1 genes in WNT4 pathway to regulate the sex differentiation in Chinese giant salamander (Andrias davidianus)
Source: Front Mol Biosci. 2022 Sep 20;9:974348. doi: 10.3389/fmolb.2022.974348 (PMC9530786; doi:10.3389/fmolb.2022.974348)

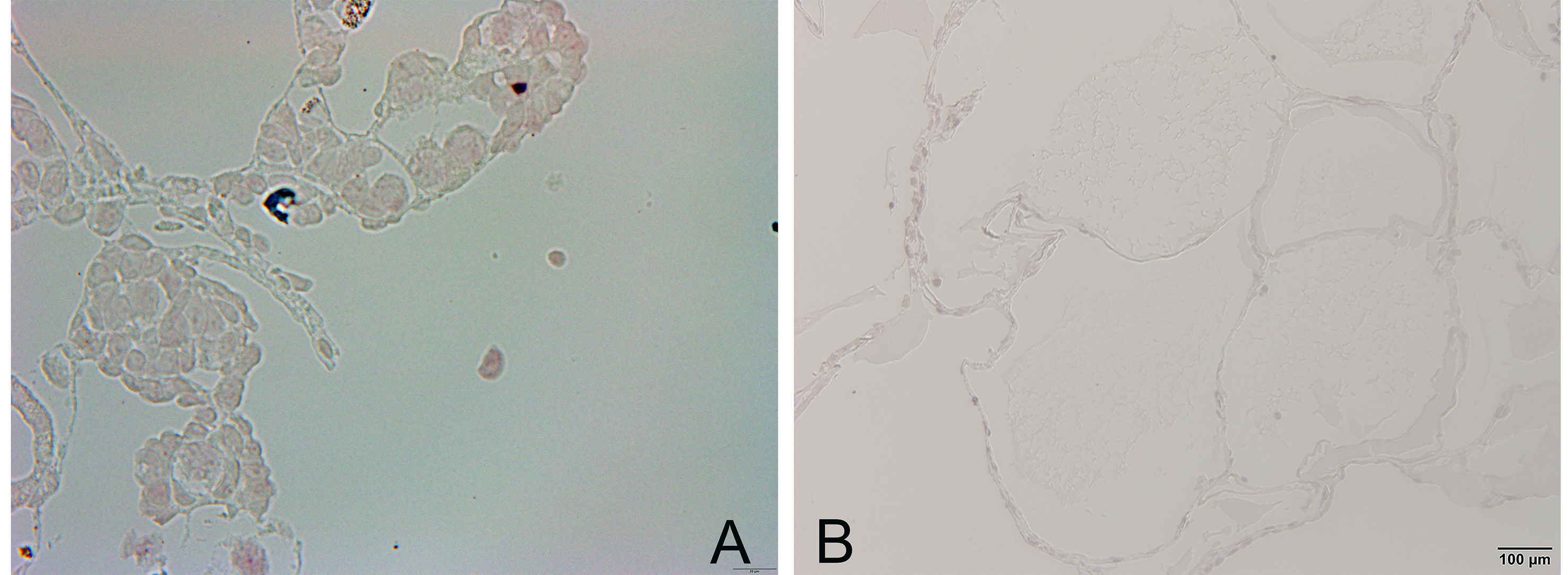

Supplement: Supplementary file 1 [file Image3.TIF]

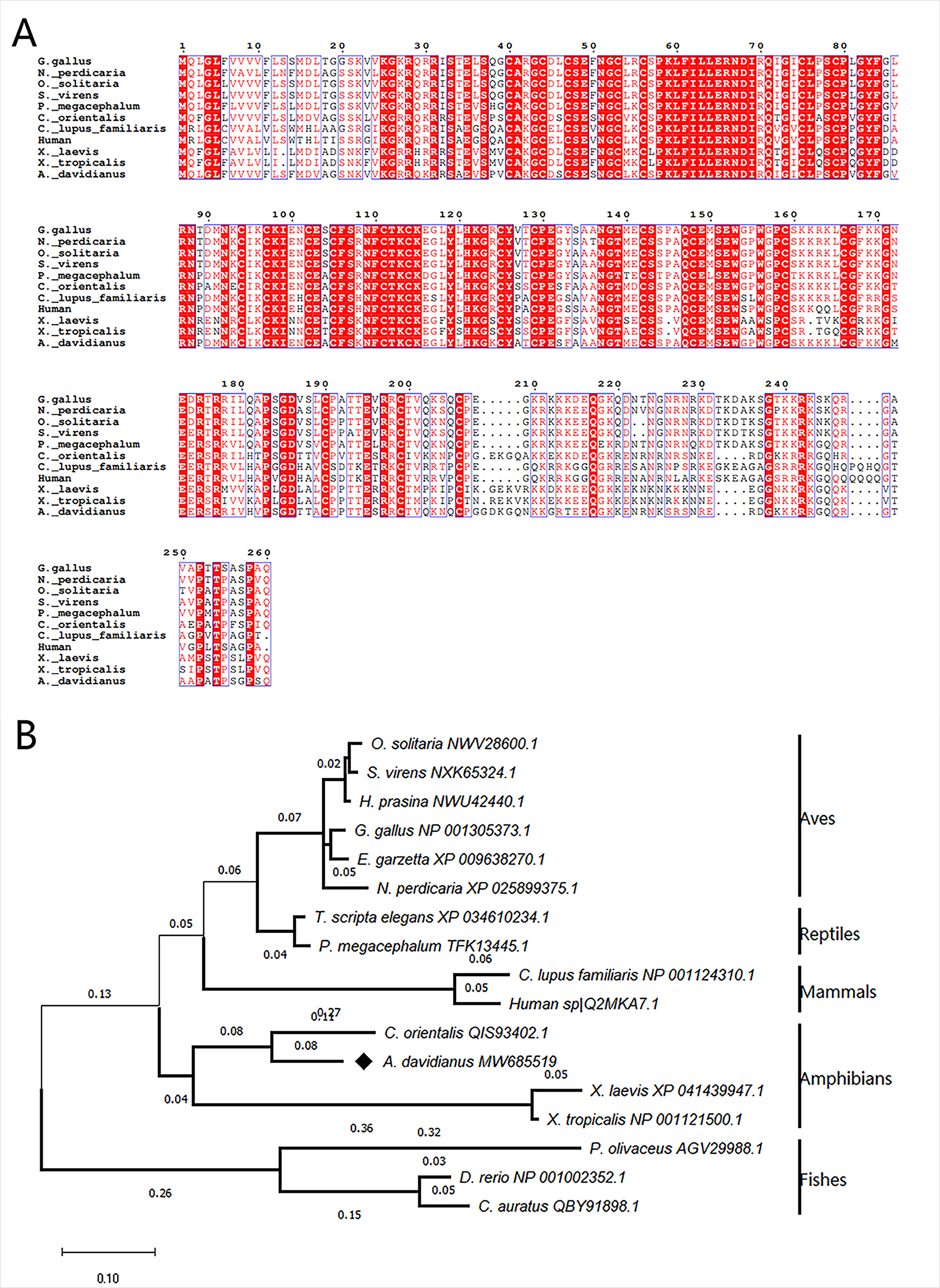

Supplement: Supplementary file 2 [file Image2.TIF]

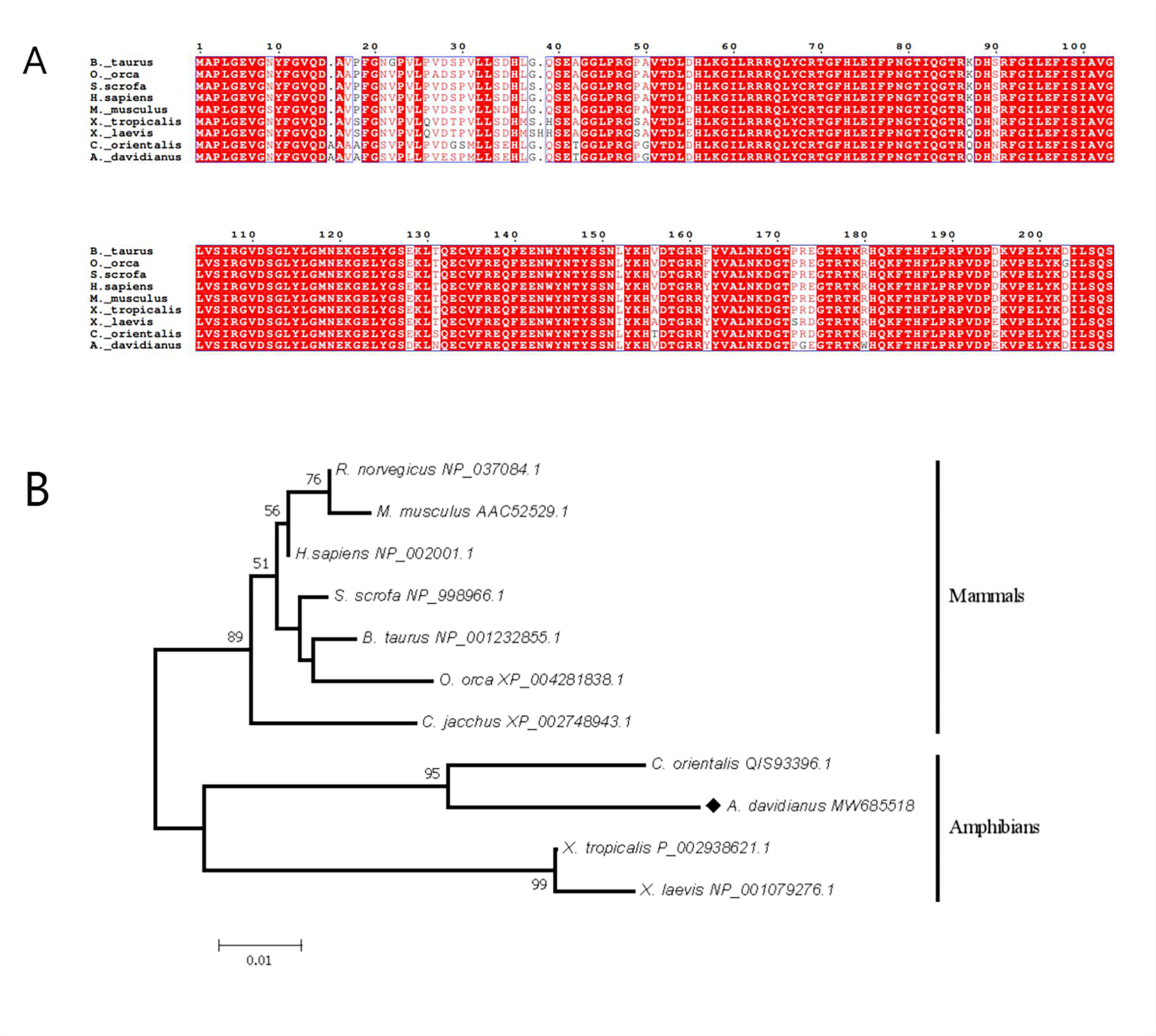

Supplement: Supplementary file 3 [file Image1.TIF]
