# Supplementary material for: Potential antagonistic relationship of fgf9 and rspo1 genes in WNT4 pathway to regulate the sex differentiation in Chinese giant salamander (Andrias davidianus)
Source: Front Mol Biosci. 2022 Sep 20;9:974348. doi: 10.3389/fmolb.2022.974348 (PMC9530786; doi:10.3389/fmolb.2022.974348)
Supplement: Supplementary file 4 [file Table1.DOC]

Dear Editor,

We would like to submit the enclosed manuscript entitled " **Potential antagonistic relationship of fgf9 and rspo1 genes in WNT4 pathway to regulate the sex differentiation in Chinese Giant Salamander (*Andrias davidianus*)**", which we wish to be considered for publication in **Frontiers in Molecular Biosciences**. No conflict of interest exits in the submission of this manuscript, and manuscript is approved by all authors for publication. I would like to declare on behalf of my co-authors that the work described was original research that has not been published previously, and not under consideration for publication elsewhere, in whole or in part. All the authors listed have approved the manuscript that is enclosed.

Based on a previous study, we first cloned the *fgf9* and *rspo1* genes using RACE. Then we characterized the expression profile of *fgf9* and *rspo1* genes in various tissues and developing gonads. Results showed that *fgf9* expression level was higher in testes than in ovaries and increased from 1 y to 6 y while *rspo1* expression was higher in ovaries than in testes. *In situ* hybridization assay showed that both *fgf9* and *rspo1* genes expressed at 62 dpf in undifferentiated gonad, and *fgf9* gene was mainly expressed in spermatogonia and sertoli cells in testis while strong positive signal of *rspo1* was detected in granular cell in ovary. During sex-reversal, *fgf9* expression was significantly higher in reversed testes and normal testes than in ovaries, and opposite expression pattern was detected for *rspo1.* When FH535 was used to inhibit Wnt/β-catenin pathway, expression of *rspo1* was down-regulated. Conversely, expression of *fgf9, dmrt1, ftz-f1* and *cyp17* were up-regulated. Furthermore, when *rspo1 and fgf9* were knocked down using RNAi technology, respectively. We observed that female biased genes were down regulated in ovary primordial cells after *rspo1* was knocked down, while the opposite expression profile was observed in testis primordial cells after *fgf9* was knocked down. These results suggested that *fgf9* and *rspo1* played an antagonistic role to regulate sex differentiation in the process of the gonadal development and provided a foundation for further functional characterizations. The article was appropriate for **Frontiers in Molecular Biosciences**.

The following is a list of possible reviewers for your consideration:

1.Bao Shi, Yellow Sea Fisheries Research Institute, Chinese Academy of Fishery Sciences, Qingdao，266071, China

Email:shibao@ysfri.ac.cn

2. Wang Na, Chinese academy of fishery sciences, China.

E-mail:wangna@ysfri.ac.cn

3.Feng He, Ocean University of China, China

E-mail. [hefengouc@ouc.edu.cn](mailto:hefengouc@ouc.edu.cn)

4. Xiangshan Ji, Shandong Agricultural University, Taian, China

Email:xsji@sdau.edu.cn

We deeply appreciate your consideration of our manuscript, and we look forward to receiving comments from the reviewers. If you have any queries, please don’t hesitate to contact me at the address below.

Thank you and best regards.

Yours sincerely,

Qiaomu Hu, Ph.D.(email: hqmu0806@163.com)

Yangtze River Fisheries Research Institute, Chinese Academy of Fishery Sciences, Wuhan, Hubei 430223, China
